# Real-time quantitative PCR assay development and application for assessment of agricultural surface water and various fecal matter for prevalence of *Aliarcobacter faecis* and *Aliarcobacter lanthieri*

**DOI:** 10.1186/s12866-020-01826-3

**Published:** 2020-06-16

**Authors:** Mary G. Miltenburg, Michel Cloutier, Emilia Craiovan, David R. Lapen, Graham Wilkes, Edward Topp, Izhar U. H. Khan

**Affiliations:** 1 Ottawa Research and Development Centre (ORDC), Agriculture and Agri-Food Canada, 960 Carling Ave, Ottawa, Ontario K1A 0C6 Canada; 2 Canadian Food Inspection Agency (CFIA), Ottawa, ON Canada; 3 Natural Resources Canada, Ottawa, ON Canada; 4 London Research and Development Centre (LRDC), Agriculture and Agri-Food Canada, London, ON Canada

**Keywords:** qPCR, *Aliarcobacter faecis*, *Aliarcobacter lanthieri*, Agricultural watershed, Surface water, Assay, Fecal matter

## Abstract

**Background:**

*Aliarcobacter faecis* and *Aliarcobacter lanthieri* are recently identified as emerging human and animal pathogens. In this paper, we demonstrate the development and optimization of two direct DNA-based quantitative real-time PCR assays using species-specific oligonucleotide primer pairs derived from *rpoB* and *gyrA* genes for *A. faecis* and *A. lanthieri*, respectively. Initially, the specificity of primers and amplicon size of each target reference strain was verified and confirmed by melt curve analysis. Standard curves were developed with a minimum quantification limit of 100 cells mL^− 1^ or g^− 1^ obtained using known quantities of spiked *A. faecis* and *A. lanthieri* reference strains in autoclaved agricultural surface water and dairy cow manure samples.

**Results:**

Each species-specific qPCR assay was validated and applied to determine the rate of prevalence and quantify the total number of cells of each target species in natural surface waters of an agriculturally-dominant and non-agricultural reference watershed. In addition, the prevalence and densities were determined for human and various animal (e.g., dogs, cats, dairy cow, and poultry) fecal samples. Overall, the prevalence of *A. faecis* for surface water and feces was 21 and 28%, respectively. The maximum *A. faecis* concentration for water and feces was 2.3 × 10^7^ cells 100 mL^- 1^ and 1.2 × 10^7^ cells g^− 1^, respectively. *A. lanthieri* was detected at a lower frequency (2%) with a maximum concentration in surface water of 4.2 × 10^5^ cells 100 mL^− 1^; fecal samples had a prevalence and maximum density of 10% and 2.0 × 10^6^ cells g^− 1^, respectively.

**Conclusions:**

The results indicate that the occurrence of these species in agricultural surface water is potentially due to fecal contamination of water from livestock, human, or wildlife as both species were detected in fecal samples. The new real-time qPCR assays can facilitate rapid and accurate detection in < 3 h to quantify total numbers of *A. faecis* and *A. lanthieri* cells present in various complex environmental samples.

## Highlights

1. Novel qPCR assays for *A. faecis* and *A. lanthieri*
2. Identifying prevalence of *Aliarcobacter* spp. in environmental samples
3. Quantitation of *A. faecis* and *A. lanthieri* in water and feces

## Background

Recently, the *Arcobacter* genus has been reclassified and divided into seven new genera where novel genus *Aliarcobacter* consists of eight species [1]. Of these *Aliarcobacter* species, *A. faecis* and *A. lanthieri*, isolated from human and animal fecal sources, have been identified as pathogenic bacteria [2–4]. Some of the most prevalent *Aliarcobacter* species including *A. butzleri*, *A. cryaerophilus*, and *A. skirrowii* have been identified as causative agents for human (e.g., gastroenteritis, bacteremia, and sepsis) and animal (e.g., mastitis, diarrhea, abortion, and reproductive disorders) infections [5]*.* These species have also  been isolated from a variety of food products, including chicken, beef, pork, and shellfish as well as various aquatic sources [6–10] and pose an important risk for human infection from contamination of water and food resulting from a variety of sources including livestock and poultry wastes, agricultural runoff, septic leakages, and direct or indirect inputs of wildlife fecal matter [11–14]. Therefore, there is a need to determine the degree of prevalence and identify potential sources of contamination of *A. faecis* and *A. lanthieri* in various fecal and aquatic niches.

Conventional culture-based methods for the identification and quantification of bacterial species from potentially contaminated environmental samples are traditionally slow and cannot be used for the identification of genus *Aliarcobacter* to the species-level [15]. Biochemical tests for the correct differentiation of *Aliarcobacter* spp., including *A. faecis* and *A. lanthieri* are difficult to differentiate using fastidious selective growth conditions, especially when they are present in low concentrations and in competition with other contaminants [5, 16]. Moreover, these methods may not be accurate enough to measure cell viability, as cells may enter into viable but non-culturable (VBNC) or non-viable and non-culturable (NVNC) states. For many situations, it is important to use techniques that can quantify the total number of cells, including VBNC and NVNC states, more accurately in various complex environmental niches. Non-viable or non-culturable cells of Gram-negative bacteria can potentially contaminate water by producing virulence-associated factors and toxins that can pose health risks to humans [17, 18].

Real-time quantitative PCR (qPCR) assays have provided more rapid and robust tools to detect and quantify *Aliarcobacter* spp. in pure culture, fecal, hide, food, and complex environmental samples [19–22]. None of these developed real-time qPCR assays were capable of differentiating and quantifying *A. faecis* and *A. lanthieri* directly from environmental matrices, partly because of their unknown status and/or low abundance in these niches.

Therefore, it is necessary to develop fast and accurate methods for detecting these species in complex environmental matrices, since conventional methods are not always accurate measures for recovery and may fail to detect these species when prevalent at low concentrations and when competing with other *Aliarcobacter* spp. The main objectives of this study  were to: i) develop and optimize species-specific direct real-time qPCR assays to quantitatively detect *A. faecis* and *A. lanthieri* in environmental niches; and ii) validate and apply these qPCR assays to detect, quantify, and assess the prevalence of *A. faecis* and *A. lanthieri* in agricultural surface water and fecal samples from human and animal sources.

## Results

### Optimization of species-specific real-time qPCR assays and development of standard curves

**Fig. 1:**
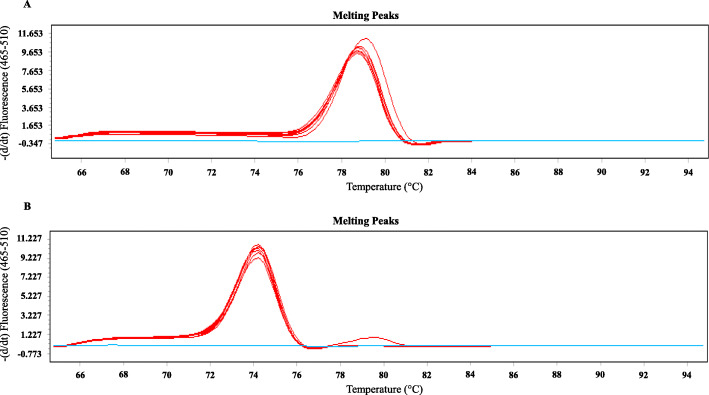
The melting curves generated for the typical amplicons showed specific relative intensity of 79 °C for *A. faecis* (*rpo*B gene) (Panel **A**) and 74 °C for *A. lanthieri* (*gyr*A gene) (Panel **B**) reference strains and field isolates against -ve controls

Two novel real-time PCR assays were developed and optimized using *A. faecis* LMG 28519 and *A. lanthieri* LMG 28516 reference strains. The assays were further validated by applying to the field isolates of each target species (*A. faecis*: *n* = 29; and *A. lanthieri*: *n* = 10). The primers specifically amplified to their target sequences with expected melting peaks of 74 °C for *A. lanthieri* and 79 °C for *A. faecis* (Fig. 1A and B) and typical amplicon sizes 152 bp and 72 bp, respectively (Figure S1A and B). Moreover, no amplification signals were observed for any other *Aliarcobacter* spp. or other bacterial reference species and strains that could potentially occur in water and fecal matter (Table 1).

**Table 1:** List of reference strains of target and other bacterial species and strains used in this study

Sr. #	Species	Source	Strain ID
1	*Haloarcobacter (Arcobacter) bivalviorum*	Shellfish	LMG 26154
2	*Aliarcobacter (Arcobacter) butzleri*	Human diarrheic stool	ATCC 49616
3	*A. cryaerophilus*	Bovine aborted fetus	NCTC 11885
4	*A. lanthieri*	Pig manure	LMG 28516
5	*A. faecis*	Human waste	LMG 28519
6	*A. skirrowii*	Lamb feces	ATCC 51322
7	*A. thereius*	Organs of aborted porcine	LMG 24486
8	*A. trophiarum*	Feces of fattening pigs	LMG 25534
9	*A. cibarius*	Broiler carcasses	LMG 21996
10	*Pseudoarcobacter (Arcobacter) defluvii*	Sewage	LMG 25694
11	*P. ellisii*	Mussels	LMG 26115
12	*P. venerupis*	Shellfish	LMG 26156
13	*Malacobacter (Arcobacter) halophilus*	Hypersaline lagoon	ATCC BAA-1022
14	*M. marinus*	Mix seawater, starfish and seaweed	LMG 25770
15	*M. molluscorum*	Mussels and oysters	LMG 25693
16	*M. mytili*	Mussels	LMG 24559
17	*Arcobacter nitrofigilis*	Roots	ATCC 33309
18	*Aeromonas allosaccharophila*	Diseased elvers	ATCC 51208
19	*A. bestiarum*	Infected fish	ATCC 51108
20	*A. caviae*	Epizootic of young guinea pigs	ATCC 15468
21	*A. hydrophila*	Ditch water	ATCC 13444
22	*A. jandaei*	Human feces	ATCC 49568
23	*A. media*	Marine fish	CDC 0435–84
24	*A. popoffi*	Drinking water production plant	BAA-243
25	*A. salmonicida*	Freshwater	CDC 0434–84
26	*A. schubertii*	Skin	ATCC 43700
27	*A. sobria*	Sludge	ATCC 35994
28	*A. trota*	Human feces	ATCC 49658
29	*A. veronii*	Red-leg frog	ATCC 9071
30	*A. bv. veronii*	Amputation Wound	ATCC 35625
31	*Campylobacter jejuni*	Human feces	ATCC 33291
32	*C. jejuni*	Human feces	ATCC 29428
33	*C. jejuni*	Human feces	ATCC 33291
34	*C. jejuni*	Human feces	ATCC 33292
35	*C. jejuni* subsp. *doylei*	Human feces	ATCC 49349
36	*C. coli*	Swine	ATCC 43136
37	*C. coli*	–	ATCC 49941
38	*C. coli*	Marmoset feces	ATCC 43478
39	*C. lari*	Human feces	ATCC 43675
40	*C. helveticus*	Cat	ATCC 51210
41	*C. fetus subsp. fetus*	Blood	ATCC 15296
42	*C. hyointestinalis*	Intestine of swine	ATCC 35217
43	*C. lanienae*	–	CCUG 44467
44	*C. upsaliensis*	Dog feces	ATCC 43954
45	*Escherichia coli* O157:H7	Environmental isolate	–
46	*E. coli*	Canine	ATCC 35218
47	*Enterococcus avium*	Clinical isolate	ATCC 49464
48	*E. casseliflavus*	–	ATCC 700327
49	*E. durans*	Human feces	ATCC 6056
50	*E. faecalis*	Meat	ATCC 7080
51	*E. faecium*	Human feces	ATCC 6569
52	*E. gallinarum*	Chicken intestines	ATCC 49573
53	*E. hirae*	–	ATCC 8043
54	*E. saccharolyticus*	Straw bedding	ATCC 43076
55	*Pseudomonas shigelloides*	Environmental isolate	–
56	*Salmonella enterica subsp. arizonae*	–	ATC C 13314
57	*S. enterica* subsp. *diarizonae*	–	ATCC 12325
58	*S. enterica* subsp. *houtenae*	–	ATCC 29932
59	*Helicobacter pylori*	Human gastric antrum	NCTC 11637
60	*H. typhlonius*	Human caecum	CCUG 48335
61	*H. ganmani*	Intestines of mice	CCUG 43526
62	*H. marmotae*	Woodchuck liver	CCUG 52419
63	*H. cetorum*	Baluga whale feces	ATCC BAA-429
64	*Klebsiella pneumoniae*	Human serotype 3	ATCC 13883
65	*Staphylococcus aureus*	Clinical isolate	ATCC 25923
66	*S. epidermidis*	Clinical isolate	ATCC 12228
67	*Streptococcus pyogenes*	Clinical isolate	ATCC 19615

**Fig. 2:**
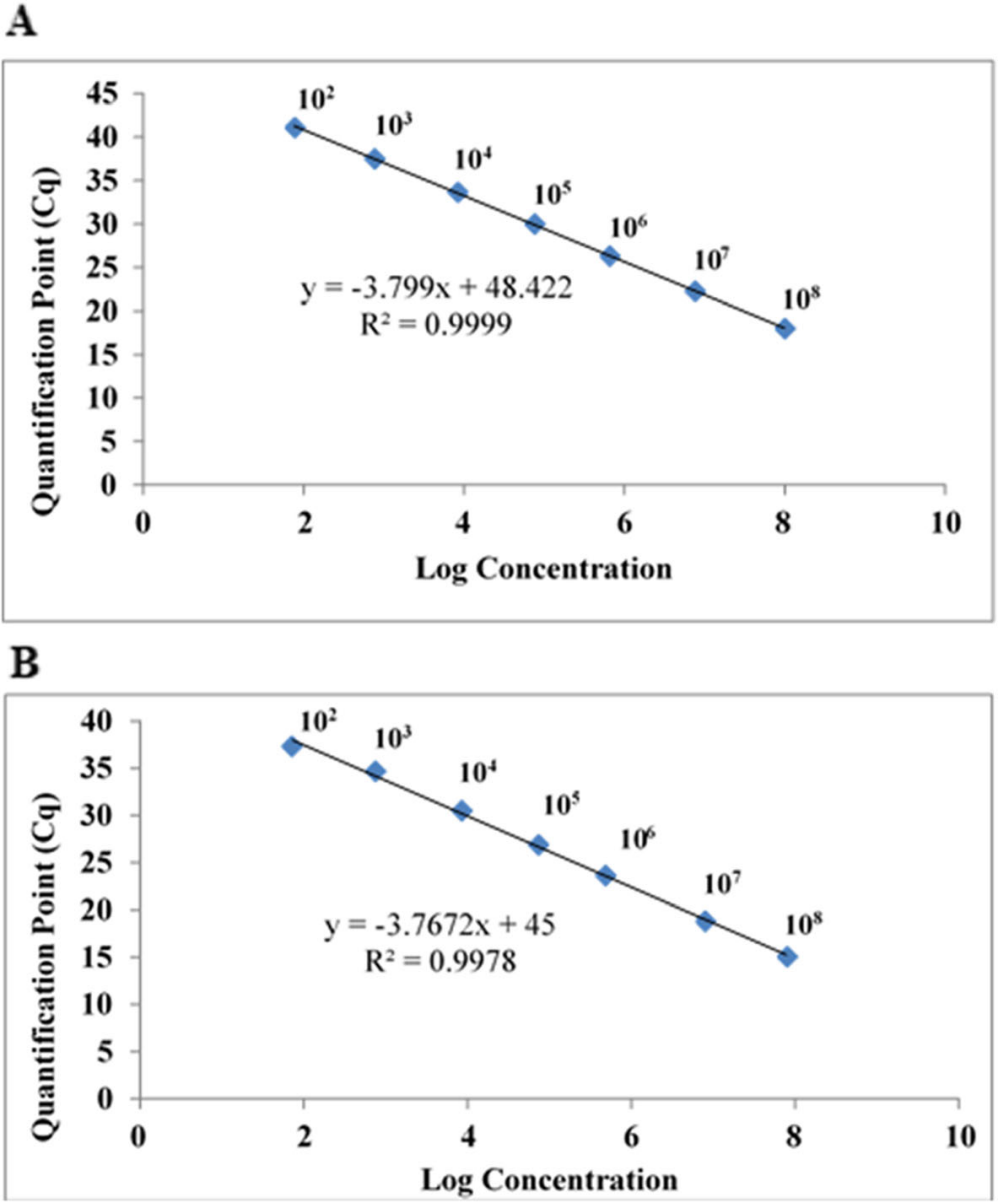
Real-time qPCR-based standard curves developed for (Panel **A**) *A. faecis* and (Panel **B**) *A. lanthieri* from 10-fold serially diluted spiked cells (from 10^2^ to 10^8^ mL^− 1^) in autoclaved agricultral surface water using species-specific qPCR assays. Each point representing the result of triplicate amplification of each dilution. The coefficients of determination R^2^ and the slopes of each regression curves are indicated. The standard curves obtained by correlation of the Quantification Cycle (Cq) values and log_10_ cell number from the amplification plot

**Fig. 3:**
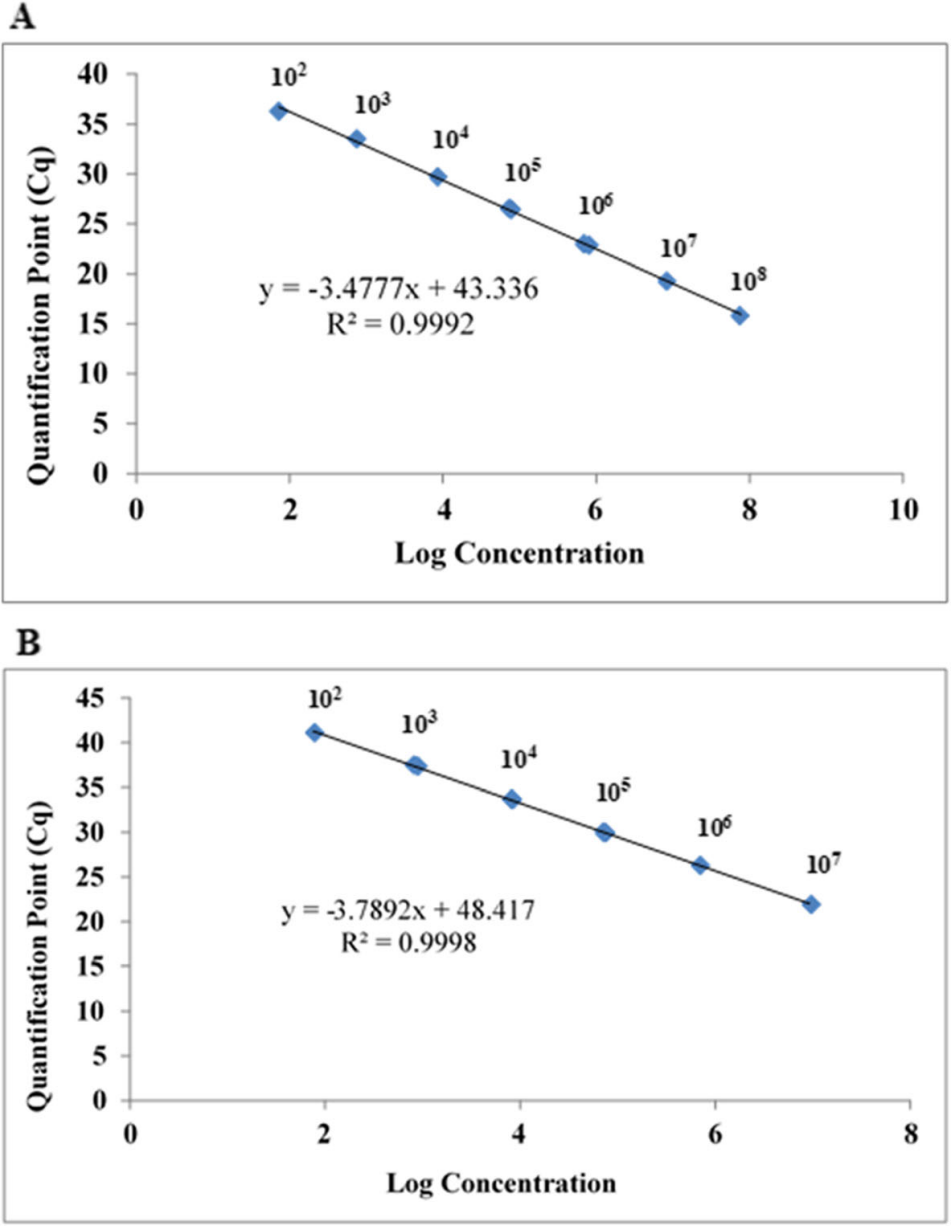
Standard curves developed from DNA extracted from 10-fold serially diluted spiked cells of *A. faecis* (Panel **A**) (ranging from 10^2^ to 10^8^ g^− 1^) and *A. lanthieri* (Panel **B**) (ranging from 10^2^ to 10^7^ g^- 1^) in cow manure using species-specific qPCR assays. The standard curves obtained from amplification plot by correlation of the Quantification Cycle (Cq) cycle values and number of cells g^− 1^ of feces. Each point is representing the result of duplicate amplification of each dilution where the correlation coefficients R^2^ and the slopes of the regression curve are shown

The limit of detection for quantitative analysis of each optimized real-time PCR assay was determined by developing standard curves of reference strains of *A. faecis* and *A. lanthieri* DNA templates extracted from spiked water and dairy cow manure samples, in units of number of cells mL^− 1^ (Fig. 2A and B) and cells g^− 1^ (Fig. 3A and B). Although a minimum of 10 cells mL^− 1^ or 10 cells g^− 1^ were also used for the quantitative assay, amplification was observed at ≥40 Cq value; therefore, Cq value ≥42 thresholds were considered as negative or indeterminate.

### qPCR assay validation and application for detection and quantitation of *A. faecis* and *A. lanthieri* in agricultural surface water and fecal sources

The qPCR assays were further validated and applied by analysing a total of 804 environmental (fecal and surface water) samples. Of the total 588 agricultural surface water samples, *A. faecis* was detected at a frequency of 21% (*n* = 124), while *A. lanthieri* (*n* = 13) was only detected in 2% of samples. Similarly, of the total 216 (human, *n* = 19; animals, *n* = 197) fecal samples, *A. faecis* (*n* = 61) was detected more commonly (28%) than *A. lanthieri* (10%; *n* = 22).

Further comparative analyses showed that the rate of *A. faecis* prevalence was significantly higher (*p* < 0.05) than *A. lanthieri* in agricultural sites (Table 2). Interestingly, only *A. faecis* (19% frequency), not *A. lanthieri*, was detected at the reference sampling site 24 (Table 2). Overall, the frequency of co-occurrence of these two target species was low and observed in only one single surface water sample, which was collected from an agricultural drainage ditch with upstream proximity to dairy livestock operations. Moreover, among the 11 agriculturally impacted sites, *A. faecis* was detected at a significantly (*p* < 0.05) higher frequency (> 20%) at sampling sites 5, 6, 10, 18 and 20 as compared to sampling sites 1, 8, 9, 19, 21 and 253 (< 20%). There was no significance (*p* > 0.05) difference  in the occurrence of *A. lanthieri* among the sites.

**Table 2:** Total number (588) of surface water samples collected from the South Nation River watershed tested for detection and prevalence of *A. faecis* and *A. lanthieri*

Sampling sites	Total samples	No. (%) of ***A. faecis***	No. (%) of ***A. lanthieri***
1	30	5 (17)	1 (3)
5	76	19 (25)	0
6	76	24 (32)	1 (1)
8	34	3 (9)	0
9	33	3 (9)	2 (6)
10	42	12 (29)	0
18	75	19 (25)	4 (5)
19	28	2 (7)	0
20	67	14 (21)	1 (1)
21	26	4 (15)	3 (12)
253	27	5 (19)	1 (4)
24	74	14 (19)	0

Of the total 216 fecal samples collected from human and various animal fecal sources, 28% (*n* = 61) and 10% (*n* = 22) samples were positive for *A. faecis* and *A. lanthieri*, respectively. Among these different fecal samples, *A. faecis* was detected at higher frequencies in human, cat, cow, dog, and pig, compared to *A. lanthieri* which was detected at lower frequency (Table 3). Interestingly, only one fecal sample from chicken was positive for *A. lanthieri* whereas one fecal sample from sheep was positive for *A. faecis* . On the other hand, duck, goat, and pony fecal samples were negative for both target species. Similar to the water samples, a low frequency of co-occurrence of both species in only four (cow: *n* = 2; human: *n* = 1; pig: *n* = 1) fecal samples was observed. Additional comparative analysis showed, overall, no significant (*p* > 0.05) difference  in the rate of prevalence of *A. faecis* and *A. lanthieri* between human and animal fecal samples was observed. Similarly, no significant difference between the rate of prevalence of *A. faecis* and *A. lanthieri* was found among human, cat, and dog fecal samples. However, a significantly higher frequency of occurrence (*p* < 0.05) of *A. faecis* than *A. lanthieri* was observed between cow and pig fecal samples.

**Table 3:** Total number (216) of human and animal fecal samples tested and positive  for *A. faecis* (61) and *A. lanthieri* (22) using species-specific qPCR assays

Fecal Sources	Total samples	No. (%) of ***A. faecis***	No. (%) of ***A. lanthieri***
Human	19	6 (31)	1 (5)
Cat	20	4 (20)	1 (5)
Chicken	8	0	1 (13)
Cow	68	14 (21)	5 (7)
Dog	18	2 (11)	1 (6)
Duck	1	0	0
Goat	4	0	0
Pig	75	34 (45)	13 (17)
Pony	2	0	0
Sheep	1	1 (100)	0

**Fig. 4:**
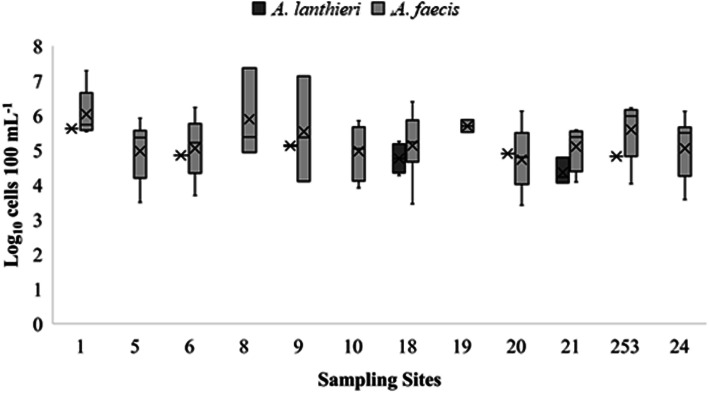
Cell concentration (Log_10_ cells 100 mL^− 1^) of *A. lanthieri* and *A. faecis* in surface water samples

Furthermore, the total cell concentrations of the 124 *A. faecis* and 13 *A. lanthieri* positive surface water samples ranged from 2.57 × 10^3^ to 2.29 × 10^7^ cells 100 mL^− 1^ and 1.15 × 10^4^ to 4.16 × 10^5^ cells 100 mL^− 1^, respectively. However, the 112 *A. faecis* positive surface water samples gave 10^3^ to 10^5^ cells 100 mL^− 1^, where only 12 of the positive samples had high concentrations (10^6^ to 10^7^ cells 100 mL^− 1^) compared to 13 *A. lanthieri* positive samples that had 10^4^ to 10^5^ cells 100 mL^− 1^. Two agricultural sampling sites (1 and 8) had similar maximal levels of *A. faecis* cell concentrations (1.9 × 10^7^ and 2.3 × 10^7^ 100 mL^- 1^) (Fig. 4). Although *A. lanthieri* was not detected in all agricultural sites, a similar average level (1 × 10^5^ cells 100 mL^− 1^) of cell concentrations was observed in sampling sites 1, 6, 9, 18, 20, 21, and 253. The cell concentrations of *A. lanthieri* were lower than *A. faecis* across all sites (Fig. 4). Results were further analyzed across sample sites: of the top 5% non-zero quantitative data from sites 1, 8, and 9, 1% of samples showed the cell concentrations above 4.1 × 10^5^, 2.1 × 10^5^, and 1.2 × 10^5^ cells 100 mL^− 1^ of *A. faecis* as compared to *A. lanthieri* where 1% of samples of site 1 had cell concentration above 1.2 × 10^4^ cells 100 mL^− 1^.

**Fig. 5:**
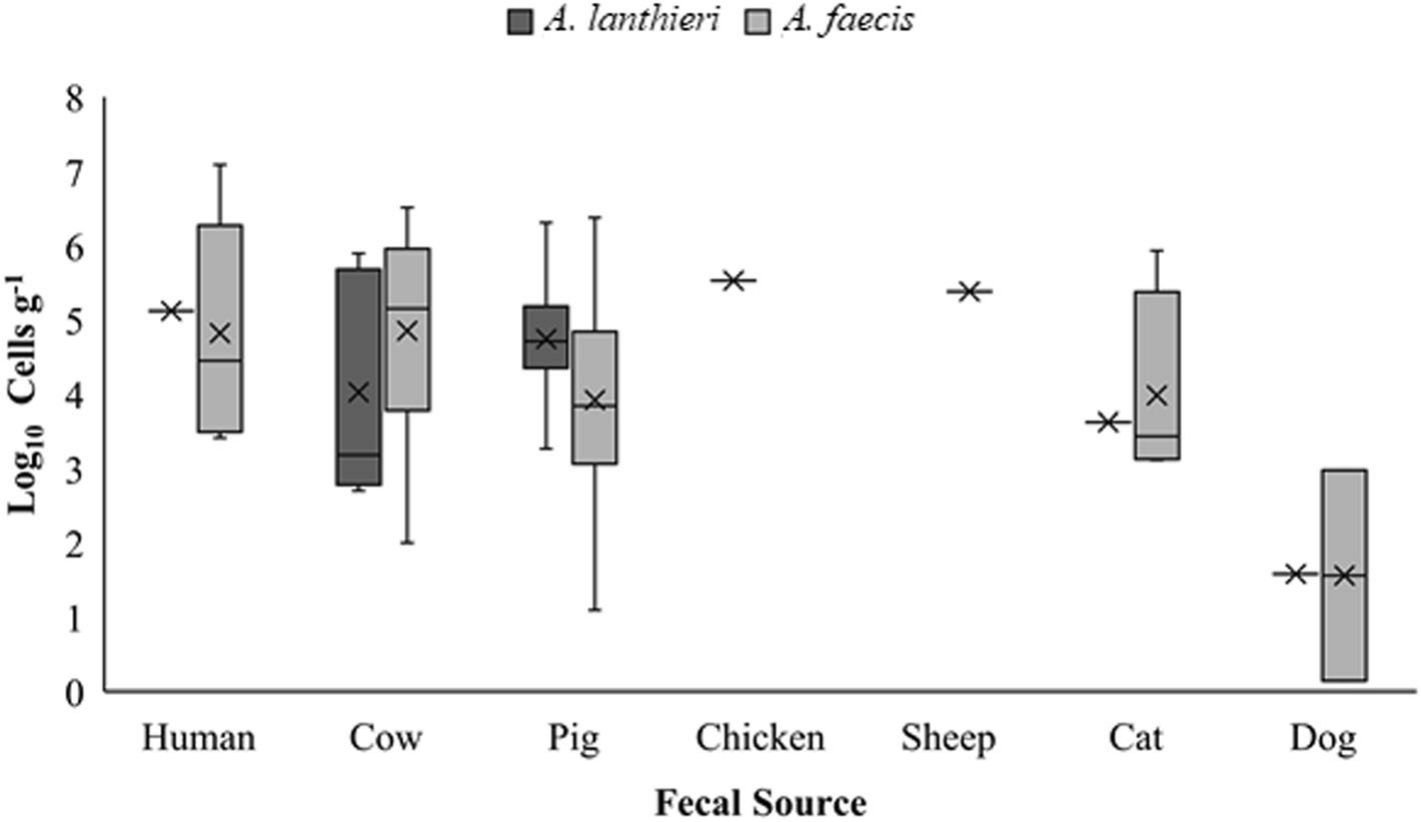
Cell concentration (Log_10_ cells g^− 1^) of *A. lanthieri* and *A. faecis* in humand and animal  fecal samples

The cell concentrations of 61 *A. faecis* and 22 *A. lanthieri* positive  fecal samples ranged from 1.4 × 10^0^ to 1.2 × 10^7^ cells g^− 1^ and 3.8 × 10^1^ to 2.0 × 10^6^ cells g^− 1^, respectively. Of the total 83 positive fecal samples for both species, 45 (54%) *A. faecis* and 21 (25%) *A. lanthieri* positive samples had cell concentration ranging from 10^3^ to 10^5^ cells g^− 1^, while six (10%) *A. faecis* and one (4%) *A. lanthieri* positive samples had higher cell concentrations in the range of 10^6^ and 10^7^ cells g^− 1^. When examining cell concentrations further across each fecal source, the highest average cell concentrations of *A. faecis* was found in human (2.3 × 10^6^ cells g^− 1^), cow (7.1 × 10^5^ cells g^− 1^), and sheep (2.4 × 10^5^ cells g^− 1^) compared to *A. lanthieri* where highest average levels were found in chicken (3.4 × 10^5^ cell g^-1^) and pig (2.7 × 10^5^ cells g^− 1^) fecal samples (Fig. 5). However, the highest cell concentration of *A. faecis* was observed in human (1.2 × 10^7^ cells g^-1^) and cow (3.3 × 10^6^ cells g^− 1^), while *A. lanthieri* had the highest cell concentrations in pig (2.0 × 10^6^ cells g^-1^) and cow (7.8 × 10^5^ cells g^− 1^) fecal samples. However, fecal samples from dog had the lowest cell concentrations for both *A. faecis* (4.8 × 10^2^ cells g^-1^) and *A. lanthieri* (3.8 × 10^1^ cells g^− 1^), respectively. Overall, of the 10% non-zero quantitative data, only 1% *A. faecis* and *A. lanthieri* positive samples had cell concentration above 2.9 × 10^6^ and 7.1 × 10^5^ cells g^− 1^, respectively.

## Discussion

Conventional culture-based multiplex PCR assays for the detection of *A. faecis* and *A. lanthieri,* along with four other closely related *Aliarcobacter* spp., were developed by Khan et al. [15]. In the present study, we further established species-specific direct DNA-based real-time quantitative PCR assays to improve the detection method for rapid identification and quantification of total number of (viable and non-viable) cells of *A. faecis* and *A. lanthieri* in surface water and fecal samples. Each species-specific qPCR assay is rapid, sensitive, and reliable for quantitative analysis of *A. faecis* and *A. lanthieri* DNA. The assay has a reproducible detection limit per reaction with linear amplification over a wide range of seven to eight orders of magnitude. qPCR assays are less time- and labor-intensive than culture-based methods, and have minimum potential for cross-contamination; therefore, the assays developed here are more robust and useful in diagnostic and analytical settings, especially when the cells of the target species are present at low concentrations [23, 24]. The other advantage is that these assays do not require post-PCR confirmation, and possess the ability to provide quick results which are more desirable for high-throughput studies [25, 26]. In addition, the fluorescent dye SYBR Green was used in the developed assays, which is more cost-effective than fluorogenic probes. qPCR assays can also detect and quantify total (viable and non-viable) number of cells, which is important as the non-viable cells can generate human immunological responses despite these cells being incapable of causing infection. Therefore, the present qPCR assays we have developed allow quantitative detection of these species from complex environmental samples even when they are present at low levels.

To validate the newly developed assays, this study analyzed 588 water samples from an agriculturally dominated watershed and 216 samples from various fecal sources, and *A. lanthieri* and *A. faecis* were detected and quantified. Overall, we found that *A. faecis* was more prevalent and occurred at higher levels than *A. lanthieri* in both fecal and water samples. Of the SNR water sites sampled, sites 1, 8, and 9 had significantly higher levels of *A. faecis* compared to sites 1 and 18 where higher relative levels of *A. lanthieri* were detected. Site 1, a drinking water intake source site, and site 8 are located on the main South Nation River stem, compared to sites 9 and 18 which are located on small stream orders that are closer to livestock operations. Although occurrence of *A. lanthieri* was not significantly different across the water sampling sites, it was most prevalent at site 21, a small agricultural drainage ditch [27] where potential fecal inputs from adjacent farm lands and wildlife  can occur readily due to tile drainage and surface runoff [28].

Levican et al. [29] found that cell counts for adhesion and invasion of different *Aliarcobacter* spp. were possible above the limits of 1.7 × 10^4^ CFU mL^− 1^ and 1.7 × 10^2^ CFU mL^- 1^, respectively. The cell concentrations of *A. lanthieri* and *A. faecis* that we detected here ranged as high as 10^7^ cells 100 mL^− 1^. Our findings are in congruence with a previous study [30] where a comparable range of concentration (2.0 × 10^5^ to 1.2 × 10^9^ cells 100 mL^− 1^) of *Arcobacter* spp. in various water sources was reported.

In order to compare the rate of prevalence of these species in agriculture and non-agricultural surface waters, site 24 was chosen as a reference site, as it is not impacted by any known direct anthropogenic activity [31]. However, *A. faecis* was detected at this site which suggests that there may be alternate sources of water contamination, possibly from wildlife. However, in previous studies human-specific bacterial markers were detected at site 24 [32, 33]. Throughout the sampling period, among agriculturally dominated SNR sites, *A. faecis* was most frequently detected at sites 5, 6, 10, 18, and 20 that have dairy operations in the upstream vicinity (Table 3). Additionally, *A. lanthieri* was most frequently detected at site 21 (Table 3), where dairy-based farming operations occur along the drainage ditch.

The prevalence of other microbial species in the SNR watershed has previously been examined, which add value in our capacity to detect *A. lanthieri* and *A. faecis* in the same study area. For example, Lyautey et al. [34] investigated the prevalence of *Listeria monocytogenes*, and also found that occurrence was associated with proximity to dairy farming operations. The authors found that sites 9 and 18 had the highest prevalence of *L. monocytogenes*. However, our results showed high frequency of *A. faecis* in site 18 compared to site 9. Frey et al. [35] detected *Campylobacter* spp. and *Salmonella* spp. at the same SNR watershed sites where cattle fecal markers were detected. *A. lanthieri* and *A. faecis* were originally isolated from human and fecal sources [4, 5], and in this study both species were detected in human and livestock feces, as well as in agricultural surface water. This strongly indicates that contamination of water by fecal matter from livestock, particularly cattle, could be linked to the prevalence of *A. lanthieri* and *A. faecis*.

## Conclusions

The qPCR assays designed here can accurately detect the prevalence and quantify the total number of cells of *A. faecis* and *A. lanthieri* in complex environmental niches. It is critical to develop alternative methods other than the widely-used culture-based techniques for the detection of gram-negative bacteria in environmental or clinical samples, as the presence of virulence, antibiotic resistance and toxin (VAT) genes can still pose a health risk even when cells are in a non-viable state. The study results suggest that routine quantitative testing of water sources for microbial contamination is important, especially in areas such as agricultural and urban communities where fecal contamination risks are higher. The developed assays could, therefore, provide rapid DNA-based tools for early and reliable detection of target species in field samples, which would help in improving water quality and intervention for reducing and eliminating the risk of contamination of *A. faecis* and *A. lanthieri* in aquatic sources.

## Methods

### qPCR assay development and optimization

#### Bacterial species and culture conditions

For testing the specificity and sensitivity of primers and real-time qPCR assays for the detection and identification of *A. faecis* and *A. lanthieri*, two reference strains of *A. faecis* LMG 28519 and *A. lanthieri* LMG 28516, were used as positive controls (Table 1). Six other *Aliarcobacter* spp., nine species from genus  *Arcobacter*, *Haloarcobacter*, *Malacobacter* and *Pseudoarcobacter*, and 50 other bacterial reference species and strains were used as negative controls (Table 1). In addition to the two LMG strains above, 29 *A. faecis* and 10 *A. lanthieri* cultures of our lab collection, isolated from various human and animal fecal and water samples, were used as positive controls. All control reference strains were grown on selective media according to appropriate aerobic and microaerophilic culture conditions. *A. faecis* and *A. lanthieri* strains were grown in Arcobacter media broth and incubated at 30 °C under microaerophilic (85% N_2_, 10% CO_2_ and 5% O_2_) conditions with continuous shaking at 125 rpm.

#### DNA extraction from pure cultures of reference strains and field isolates

The DNA from pure cultures of reference strains and field isolates was extracted using a boiling method [36] where a single colony was suspended in 75 μL TE (10 mM Tris-HCl, 1 mM EDTA, pH 8.0) buffer, boiled for 10 min and centrifuged. The supernatant containing DNA was quantified using a Qubit 3.0 fluorometer (Thermo Fisher Scientific, Waltham, MA, USA), transferred to a sterile tube, and stored at − 20 °C for further PCR analysis.

#### Spiked assay for standard curve development and quantitation

A spiking experiment was carried out to develop standard curves using *A. faecis* LMG 28519 and *A. lanthieri* LMG 28516 reference strains to assess the purity of nucleic acid in terms of yield, concentration, reproducibility and removal of potential PCR-inhibitory compounds. The experiment also helped to quantify and measure the sensitivity (least number of cells mL^− 1^) of the qPCR assays. *A. faecis* and *A. lanthieri* cells were grown under microaerophilic conditions as described above. The cells were collected by centrifugation at room temperature and re-suspended in 1 mL TE buffer. The cell concentration mL^− 1^ of each target species was measured on modified Arcobacter Agar Medium (m-AAM; Oxoid) containing selective antimicrobial agents (cefoperazone, amphotericin B, and teicoplanin) and incubated under conditions as described above. The known quantity of *A. faecis* or *A. lanthieri* reference strain cells (10^8^ cells mL^− 1^) was then simultaneously spiked and serially (10-fold) diluted from 10^8^ to 10^1^ cells mL^− 1^ in autoclaved agricultural watershed water and cow manure samples. Each spiked water sample with known cell concentration was filtered through a 0.22 μm sterile nitrocellulose filter.

Total genomic DNA was extracted from each spiked filter and 0.5 g manure sample with known cell concentration using DNeasy PowerSoil Kit (Qiagen; formerly MoBio PowerSoil DNA Isolation Kit) following the manufacturer’s instructions. The purity and concentration of DNA was measured by Qubit 3.0 fluorometer and 1% agarose gel electrophoresis using 1X TAE (0.04 M Tris-acetate, 0.001 M EDTA, pH 7.8) buffer.

#### Primer design and qPCR assay conditions

Real-time qPCR assays were developed and optimized for *A. lanthieri* by designing primer pairs from the variable region of the gyrase (*gyr*A) gene. The primers were designed based on alignment analysis of *gyr*A gene sequences of *Aliarcobacter* and other reference species and strains belong to other genera available in the GenBank database. On the other hand, the real-time PCR assay for *A. faecis* was optimized by using primers from the *rpo*B gene encoding β-subunit of RNA polymerase previously designed by Khan et al. [15].

For each target species, a SYBR Green-based species-specific monoplex real-time qPCR amplification protocol was developed and optimized with a 20 μL reaction mixture containing 10 μL SsoAdvanced EvaGreen Supermix (Bio-Rad, Hercules, CA, USA), 10–50 ng μL^− 1^ of purified DNA template of each target species, 0.01 uM forward and reverse primers (Table 4), 5% dimethyl sulfoxide (DMSO), and 0.1 uM Bovine Serum Albumin (BSA). The final volume was adjusted to 20 μL by adding sterile water.

**Table 4:** Oligonucleotide primer pairs and protocol used for optimized species-specific real-time qPCR-based assays

Species	Target Gene	Sequence 5ˊ-3ˊ	Anealing Temp (°C)	Product Size (bp)	Melting Peak (°C)	Reference
*A. faecis*	*rpo*B	Afae-F: GCT CCA GGA AGT ACA AAA GTA G Afae-R: AGG CTA GCA GCT ACT CCC	58.0	152	79	Khan et al. 2017 [15]
*A. lanthieri*	*gyr*A	Alan-F: CTT GGT GAA TTG CTT GAT GCA A Alan-R: CCA TTA AAT CAC TAG CTT CTG CT	55.0	72	74	This study

The reactions were run on a Lightcycler® 480 Instrument II (Roche, Indianapolis, IN, USA) with an initial denaturation at 98 °C for 3 min followed by 50 cycles of denaturation at 98 °C for 15 s, annealing temperatures of 58 °C for *A. faecis* and 55 °C for *A. lanthieri* for 30 s*,* and extension at 72 °C for 30 s. The amplified product obtained from each cell number was confirmed by melt curve analysis where the melting peak was 79 and 74 °C for *A. faecis* and *A. lanthieri*, respectively (Table 4). Due to expected small amplicon sizes, the amplified products were further confirmed on a 2% agarose gel matrix, stained (ethidium bromide 0.5 μg mL^− 1^) and visualized on a UV transilluminator using an Alpha Imager (Fisher Scientific) gel documentation system.

### Validation and application of qPCR assays

#### Study site description, and surface water and fecal sample collection

**Fig. 6:**
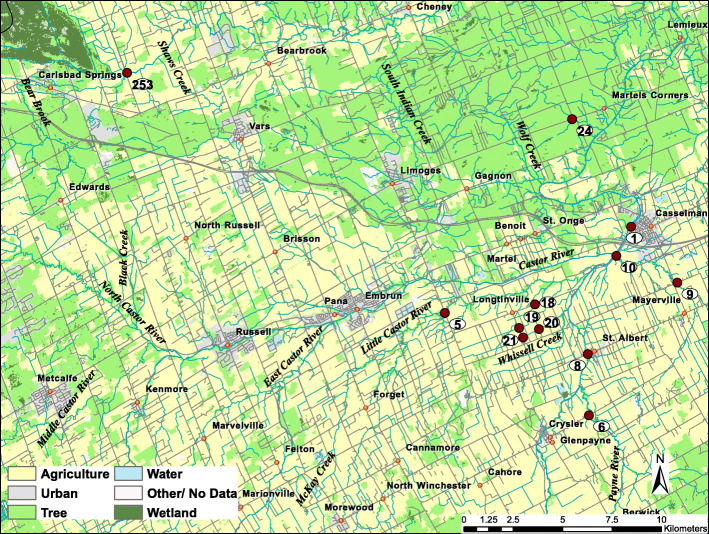
South Nation River watershed area map showing twelve sites selected for the study

The developed qPCR assays were further validated and applied to investigate the rate of prevalence and concentration of *A. faecis* or *A. lanthieri* cells in surface water and fecal samples. In order to assess the purity of total DNA in terms of removal of PCR inhibitors (such as humic acid, organic and inorganic compounds) and sensitivity of assays for quantitation of total number of cells, surface water samples were collected from the South Nation River (SNR) watershed, located near Ottawa, Ontario in eastern Canada [37]. The watershed covers an area of ~ 3900 km^2^, of which approximately 60% is used for agricultural purposes, primarily related to dairy farming. A detailed description of the watershed and sampling sites have been previously reported by Wilkes et al. [37, 38] and Lapen et al. [39] (Table 2). For this study, a total of 12 sites of varying stream orders were selected for sampling, based on their proximity to agriculturally-impacted areas. In addition, one site with no known upstream anthropogenic activity was selected as a reference site (Site 24; Edge et al.) [31] (Fig. 6). A total of 588 (from 2013 to 2018) surface water samples were collected on a bi-weekly basis between April and November. In addition, a total of 216 fecal samples from human (*n* = 19) and various animal (*n* = 197) sources including cat (*n* = 20); chicken (*n* = 8); cow (*n* = 68); dog (*n* = 18); duck (*n* = 1); goat (*n* = 4); pig (*n* = 75); pony (*n* = 2) and sheep (*n* = 1) were collected in the same region.

The surface water and fecal samples were collected in sterile polypropylene bottles and bags, placed in coolers and delivered to Agriculture and Agri-Food Canada-Ottawa, Ontario Laboratory where the samples were processed within 24 h of their collection for microbiological analysis. Water samples were filtered through 0.22 μm sterile nitrocellulose filters. The DNA from filters and fecal samples were extracted using DNeasy PowerSoil Kit and quantified by Qubit 3.0 fluorometer.

#### Quantitation of *A. faecis* and *A. lanthieri* cell concentration in environmental sources

The two optimized real-time qPCR assays described above were validated, using the developed standard curves, by detecting and quantifying the total number (viable and non-viable) of *A. faecis* and *A. lanthieri* cells 100 mL^− 1^ from agricultural surface water and fecal samples. The specificity and quality of amplified products were confirmed by analyzing and comparing the melting curves to the standard melting peaks obtained for *A. faecis* and *A. lanthieri* amplicons. In addition, the amplification quality was also validated by agarose gel electrophoresis using 100 bp DNA size marker (Thermo Fisher Scientific) (Fig. S2A&B). The gel was stained, visualized, and photographed as described in the preceding section.

### Data analysis

McNemar Chi-square Contingency and Fisher’s Exact tests were applied to compare the rate of prevalence and identify significant differences (*p* < 0.05) of *A. faecis* and *A. lanthieri* among different agricultural and non-agricultural sites, surface water and fecal samples using STATISTICA (StatSoft, Inc., 2013) [40]. 

## Supplementary information

**Additional file 1: Supplementary Figure 1A&B.** Real-time qPCR amplified product confirmation on 2% agarose gel for *A. faecis* LMG 28519 reference strain and field isolates (Panel A; Lanes 1–4) and *A. lanthieri* reference strain LMG 28516 and field isolates (Panel B; Lanes 1–4) with an expected 152 and 72 bp sizes, respectively. Lanes 5 and 11: *A. butzleri*, *A. cryaerophilus*, *A. skirrowii*, *A. thereius*, *A. trophiarum*, *A. cibarius* and no DNA template (PCR reaction mix) served as negative controls; M: 100 bp DNA size marker. **Supplementary Figure 2A&B.** Real-time qPCR amplified product confirmation on 2% agarose gel showing positive and negative field samples for *A. faecis* (Panel A) and *A. lanthieri* (Panel B) with an expected 152 and 72 bp sizes, respectively. Lane 1: *A. faecis* and *A. lanthieri* reference strains served as positive controls; M: 100 bp DNA size marker.

## Data Availability

The data generated and analyzed in this study are available upon request.

## Abbreviations

DMSO: Dimethyl sulfoxide
BSA: Bovine Serum Albumin
m-AAM: modified Arcobacter Agar Medium
SNR: South Nation River
gyrA: gyrase gene
