# Supplementary material for: Real-time quantitative PCR assay development and application for assessment of agricultural surface water and various fecal matter for prevalence of Aliarcobacter faecis and Aliarcobacter lanthieri
Source: BMC Microbiol. 2020 Jun 16;20:164. doi: 10.1186/s12866-020-01826-3 (PMC7298852; doi:10.1186/s12866-020-01826-3)
Supplement: Supplementary file 1 — Additional file 1: Supplementary Figure 1A&B. Real-time qPCR amplified product confirmation on 2% agarose gel for A. faecis LMG 28519 reference strain and field isolates (Panel A; Lanes 1–4) and A. lanthieri reference strain LMG 28516 and field isolates (Panel B; Lanes 1–4) with an expected 152 and 72 bp sizes, respectively. Lanes 5 and 11: A. butzleri, A. cryaerophilus, A. skirrowii, A. thereius, A. trophiarum, A. cibarius and no DNA template (PCR reaction mix) served as negative controls; M: 100 bp DNA size marker. Supplementary Figure 2A&B. Real-time qPCR amplified product confirmation on 2% agarose gel showing positive and negative field samples for A. faecis (Panel A) and A. lanthieri (Panel B) with an expected 152 and 72 bp sizes, respectively. Lane 1: A. faecis and A. lanthieri reference strains served as positive controls; M: 100 bp DNA size marker. [file 12866_2020_1826_MOESM1_ESM.docx]

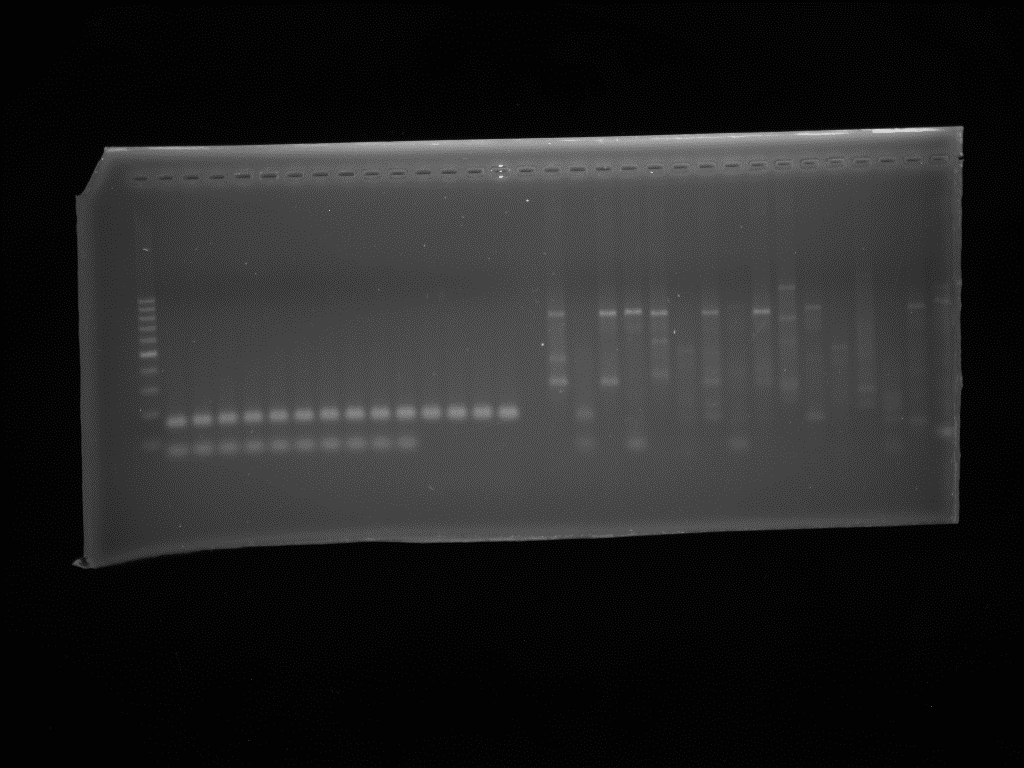

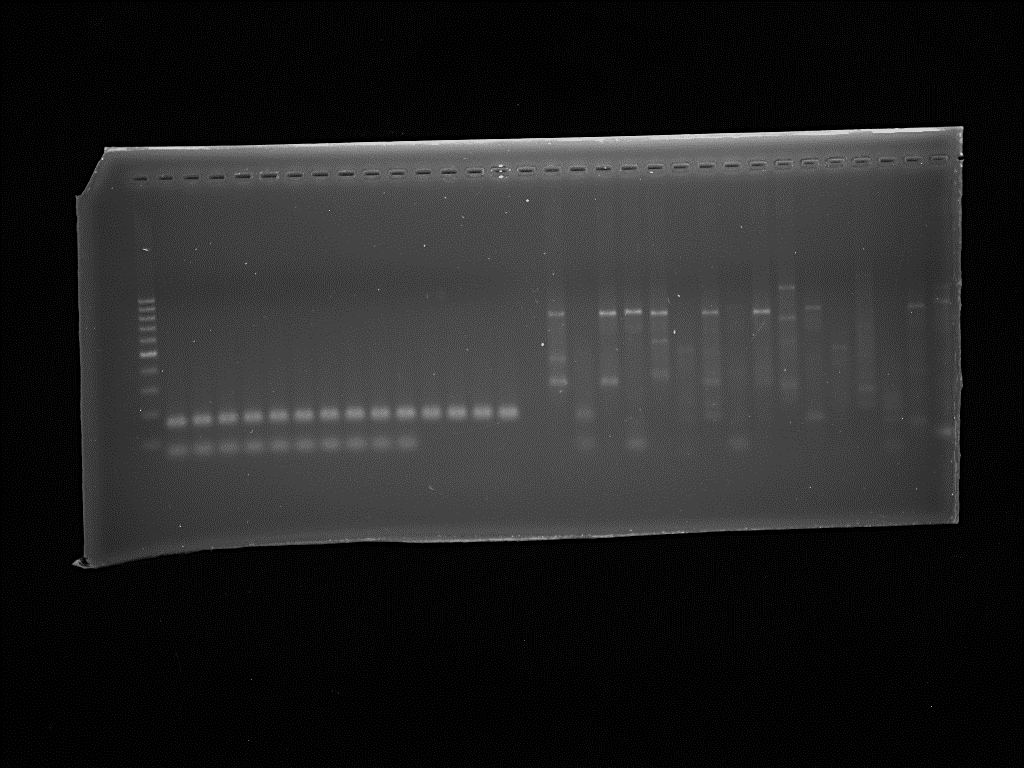

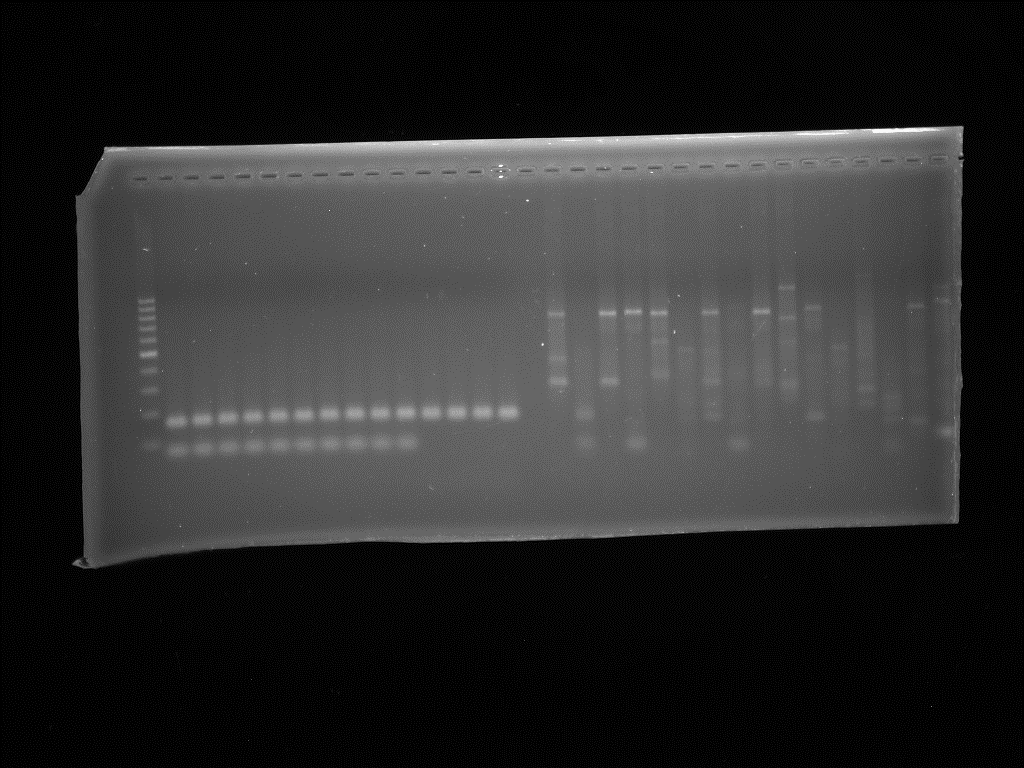

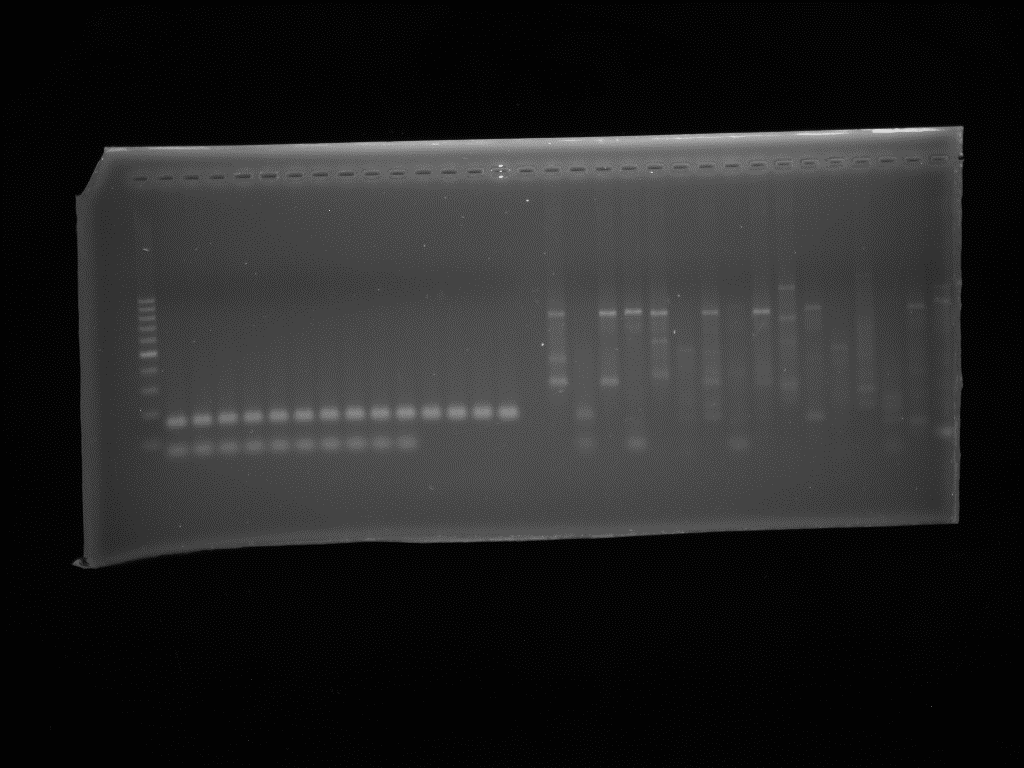

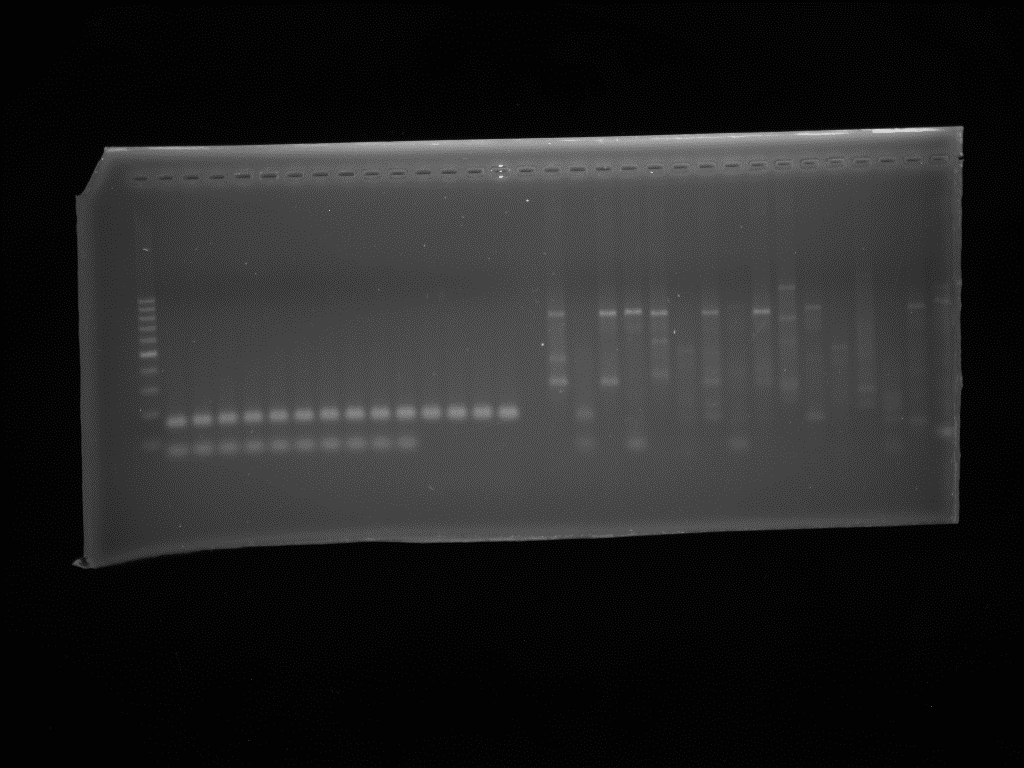

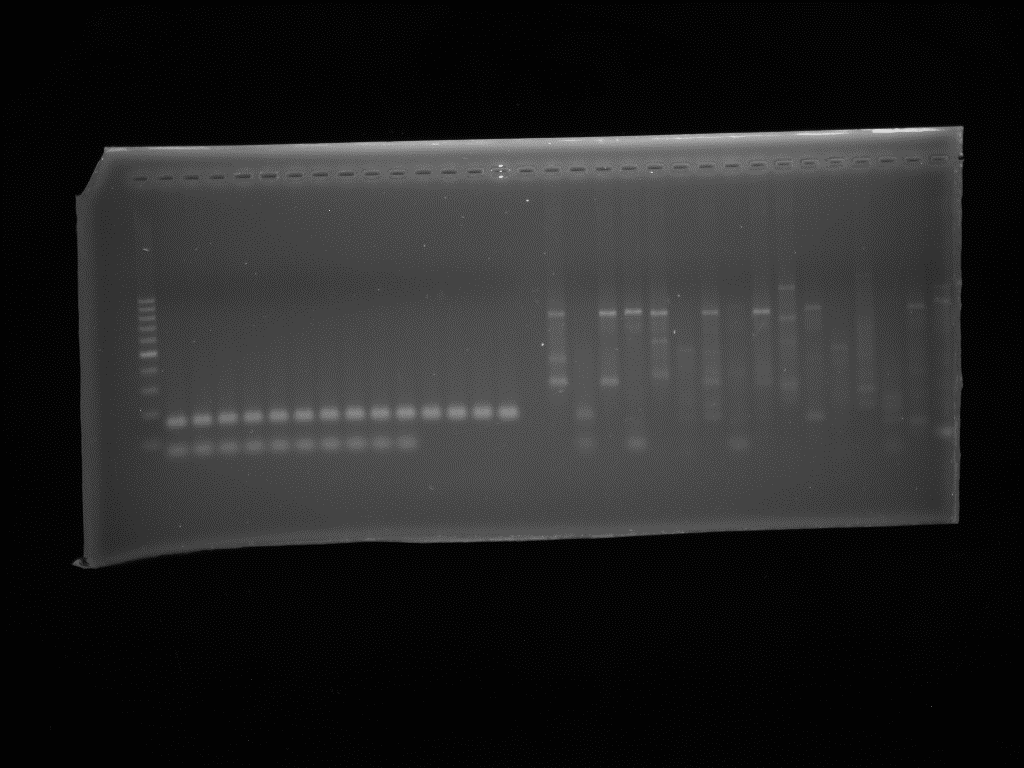

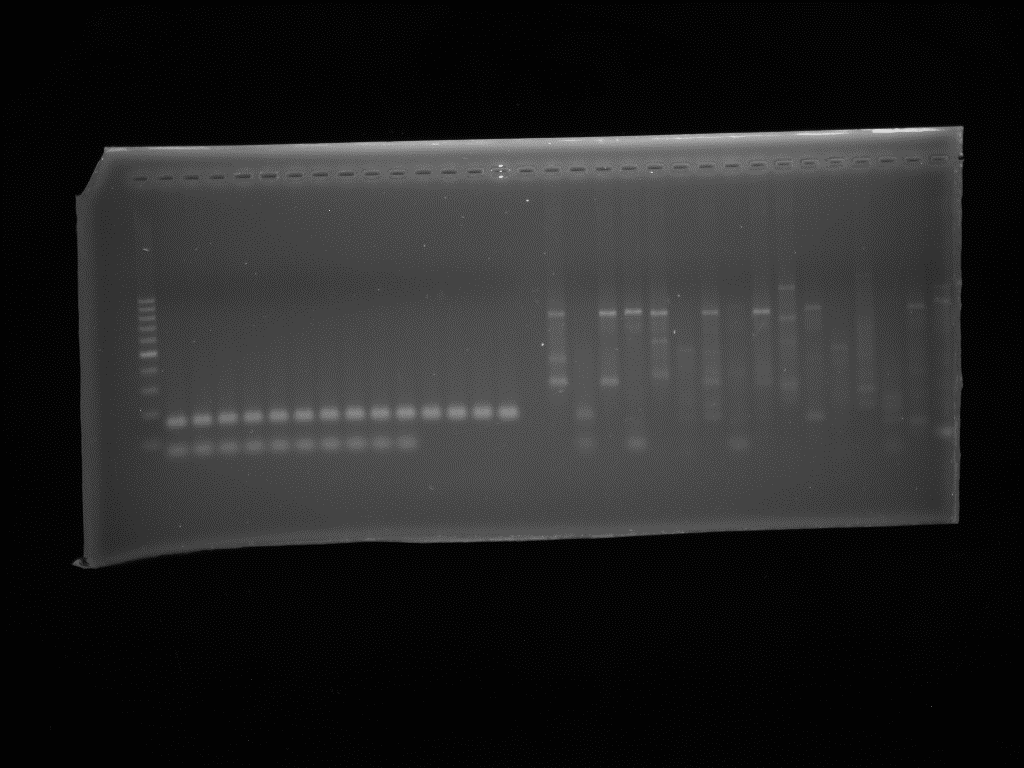

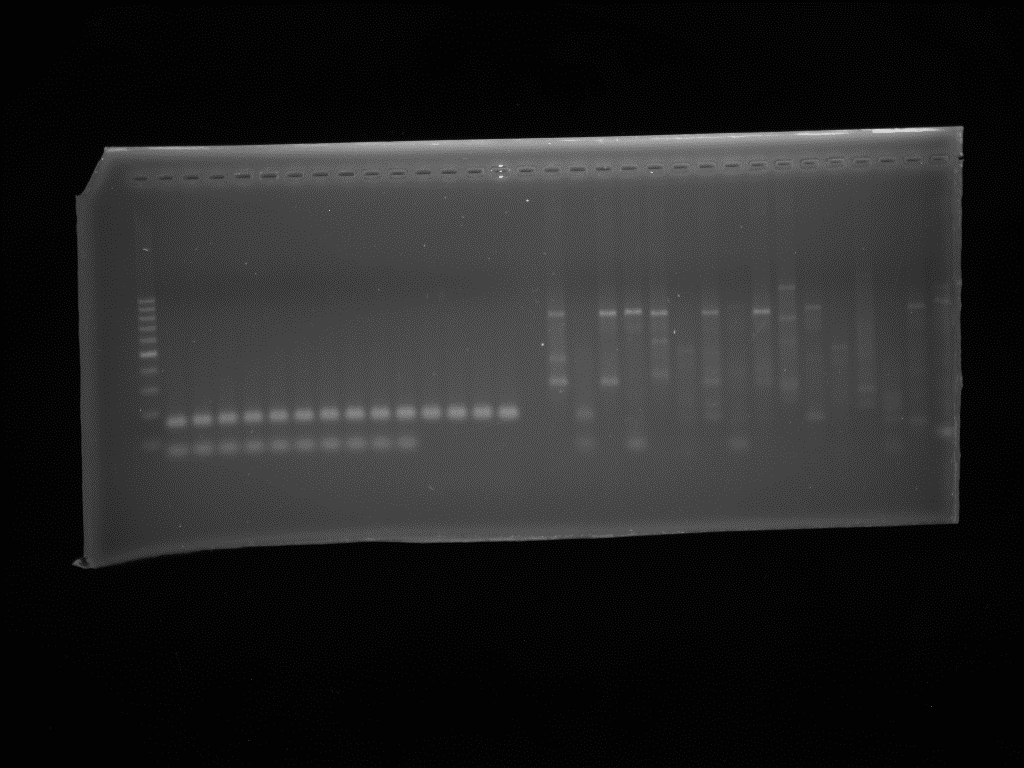


**500-**

**200-**

**800-**

**bp**

**M 1 2 3 4 5 6 7 8 9 10 11**

**A**


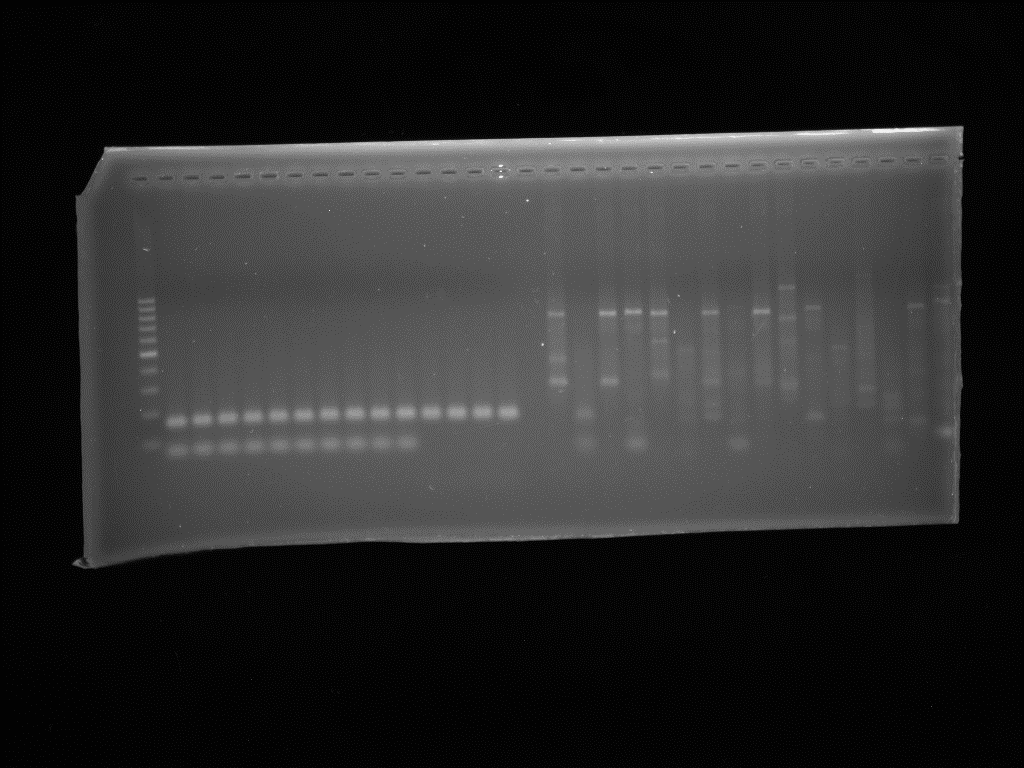

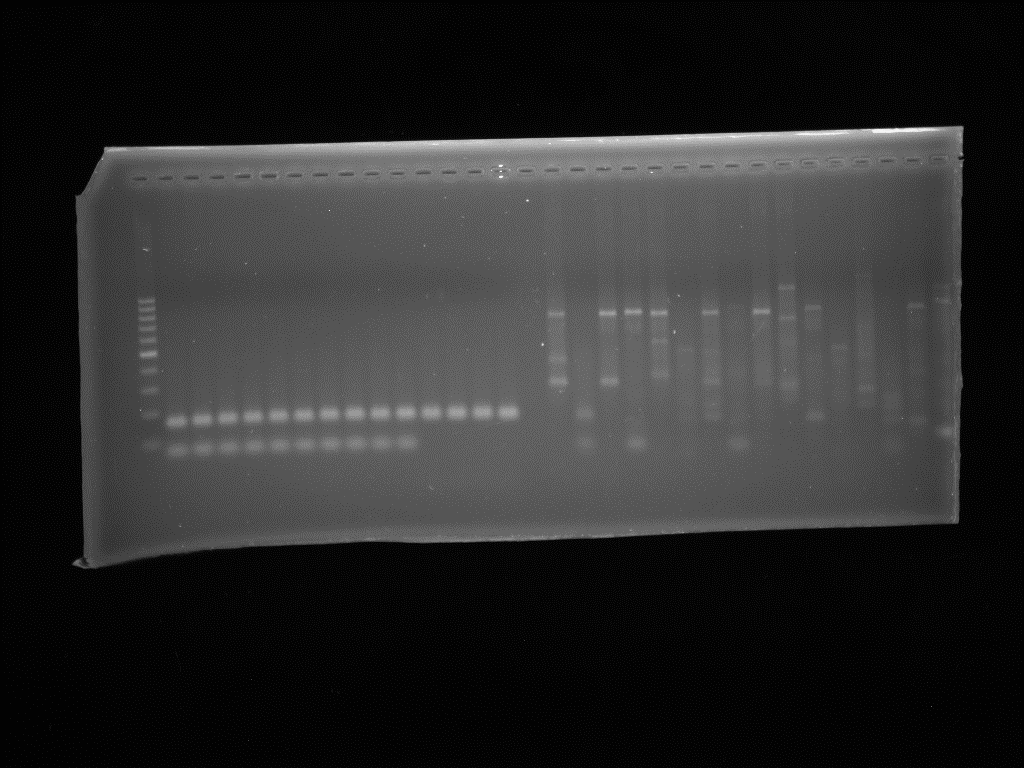

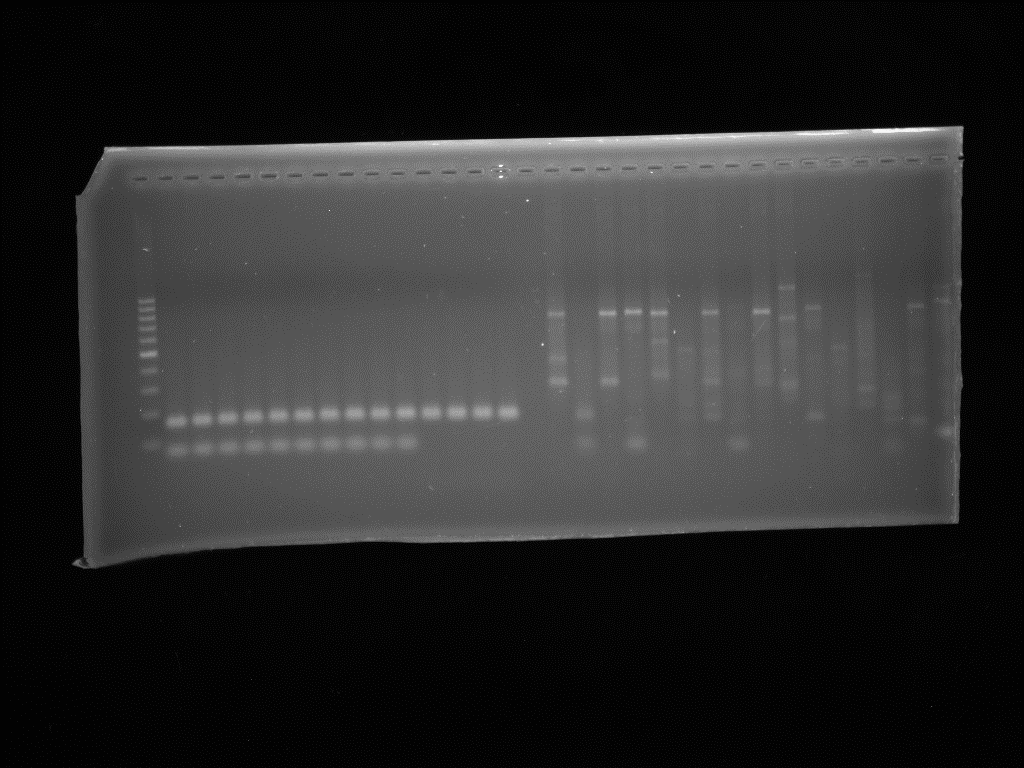

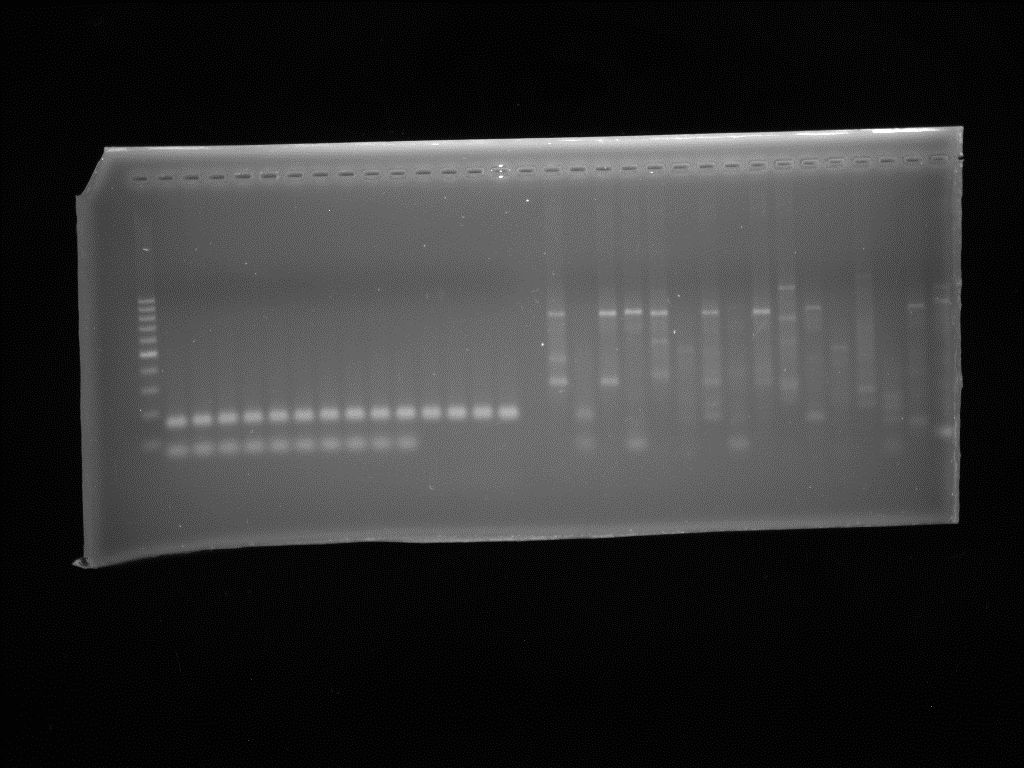


**M 1 2 3 4 5 6 7 8 9 10 11**

**500-**

**200-**

**800-**

**bp**

**B**

**Supplementary Figure 1A&B:** Real-time qPCR amplified product confirmation on 2% agarose gel for *A. faecis* LMG 28519 reference strain and field isolates (Panel A; Lanes 1-4) and *A. lanthieri* reference strain LMG 28516 and field isolates (Panel B; Lanes 1-4) with an expected 152 and 72 bp sizes, respectively. Lanes 5 and 11: *A. butzleri*, *A. cryaerophilus*, *A. skirrowii*, *A. thereius*, *A. trophiarum*, *A. cibarius* and no DNA template (PCR reaction mix) served as negative controls; M: 100 bp DNA size marker.


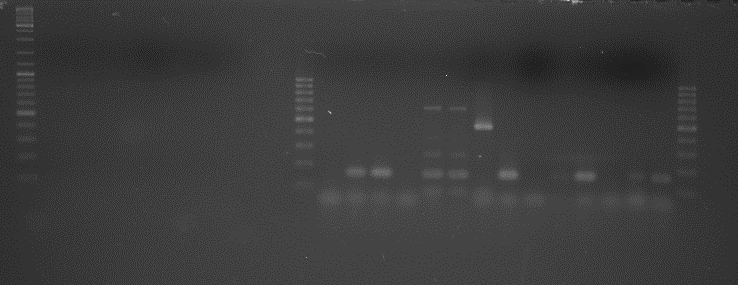

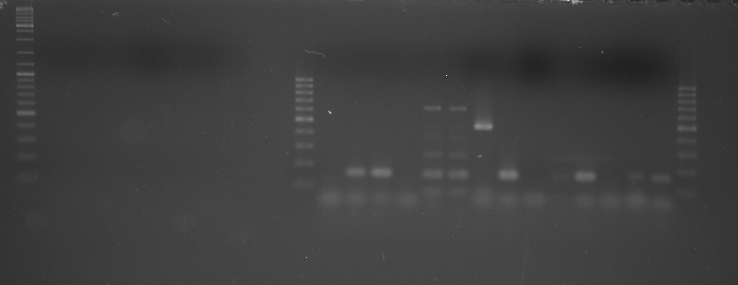

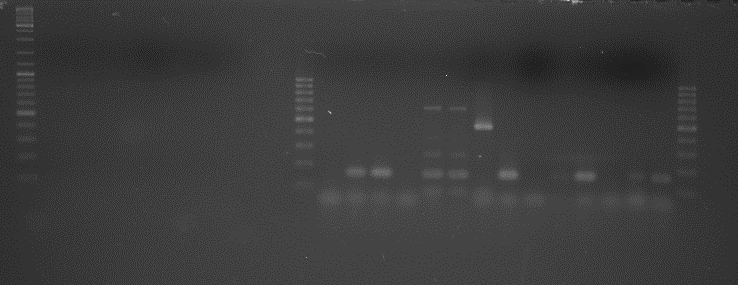

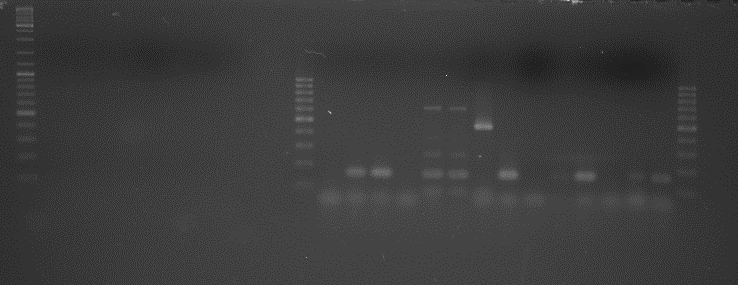


**500-**

**200-**

**800-**

**bp**

**A**

**M 1 2 3 4 5 6 7 8 9 10 11 12 13**


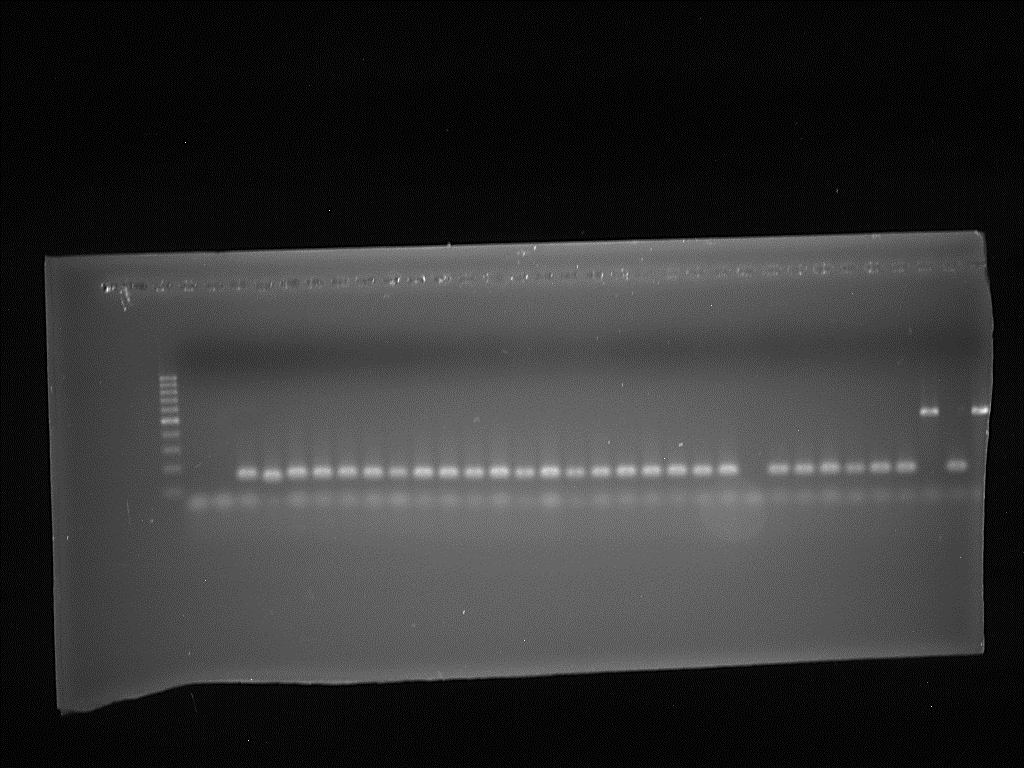

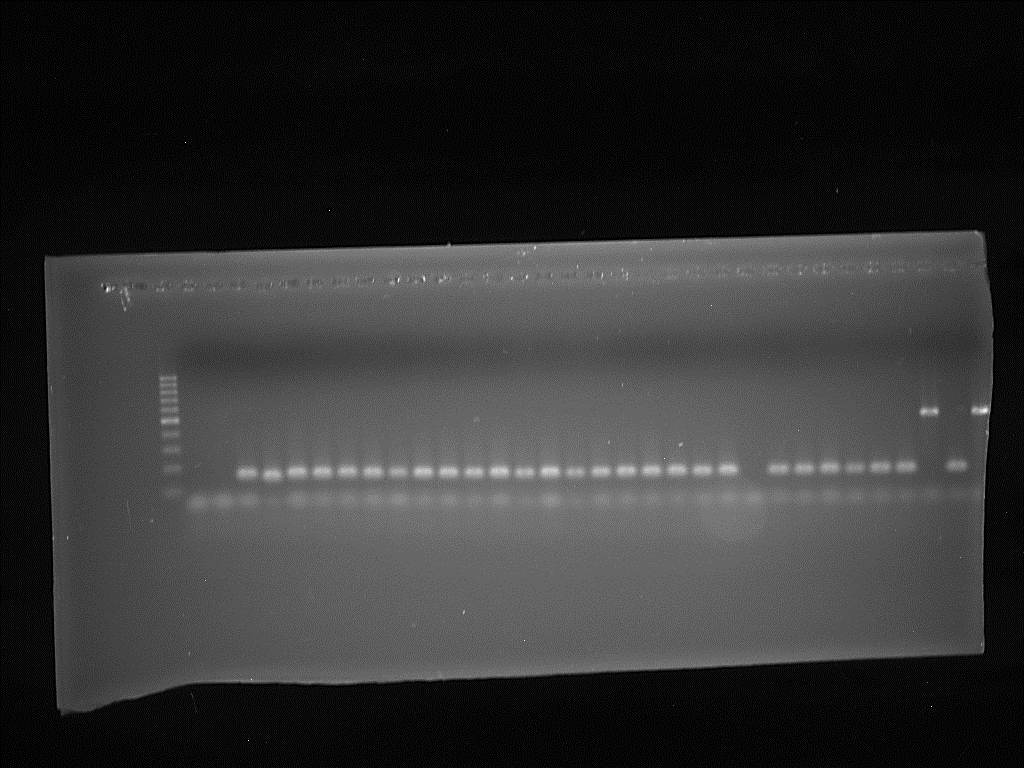

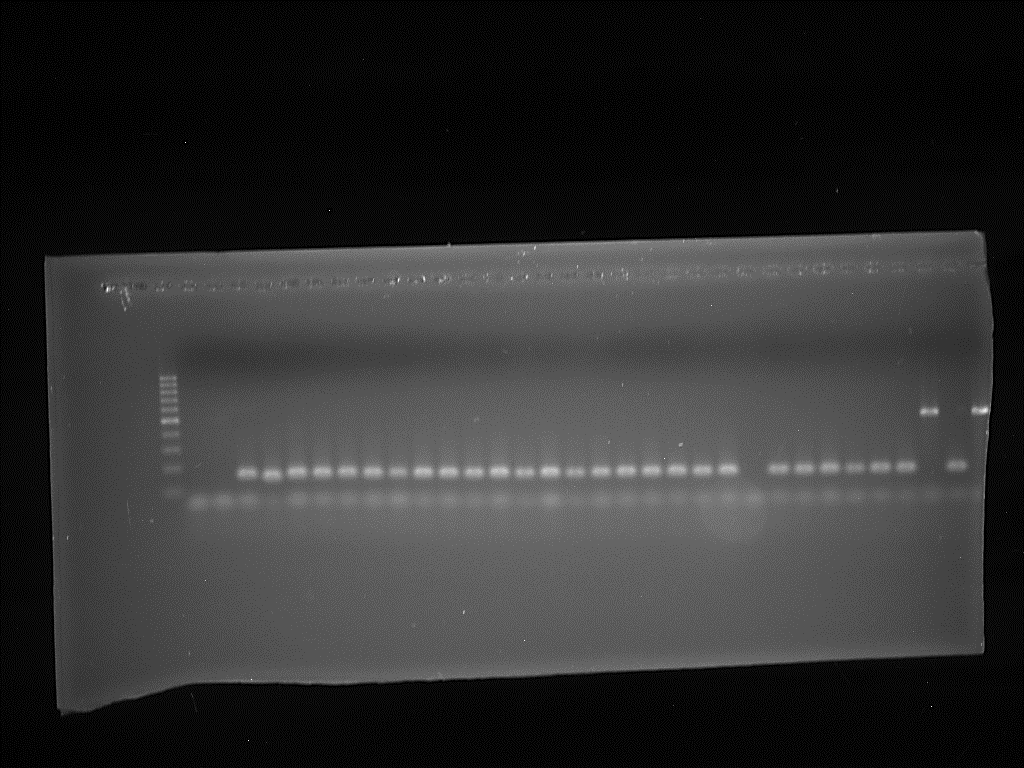

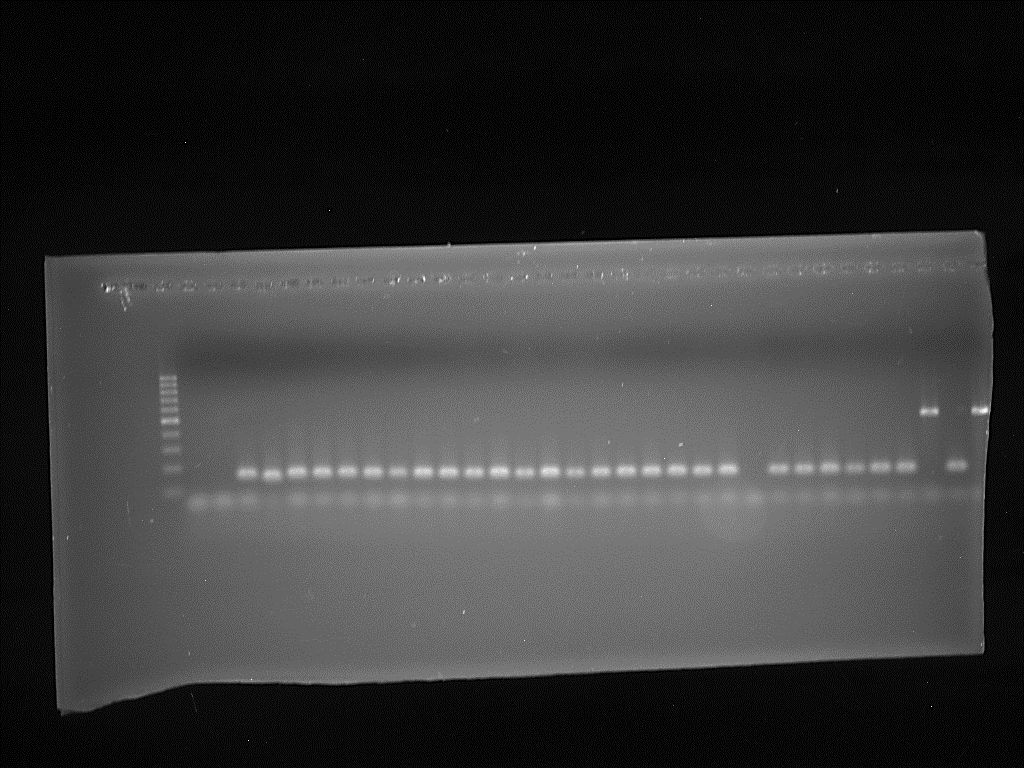


**M 1 2 3 4 5 6 7 8 9 10 11**

**500-**

**200-**

**800-**

**bp**

**B**

**Supplementary Figure 2A&B:** Real-time qPCR amplified product confirmation on 2% agarose gel showing positive and negative field samples for *A. faecis* (Panel A) and *A. lanthieri* (Panel B) with an expected 152 and 72 bp sizes, respectively. Lane 1: *A. faecis* and *A. lanthieri* reference strains served as positive controls; M: 100 bp DNA size marker.
